# Cardiac Incidentalomas in the Trauma Bay: Imaging Beyond the Red Herring

**DOI:** 10.1016/j.jaccas.2026.109221

**Published:** 2026-07-15

**Authors:** Ciaran Grafton-Clarke, Pankaj Garg

**Affiliations:** a Norfolk and Norwich University Hospitals NHS Foundation Trust, Norwich, United Kingdom; b Department of Cardiovascular and Metabolic Health, Norwich Medical School, University of East Anglia, Norwich, United Kingdom; c Department of Infection, Immunity & Cardiovascular Disease, University of Sheffield, Sheffield, United Kingdom

**Keywords:** cardiac magnetic resonance, echocardiography, mitral valve, MR sequences, pneumothorax

**Figure:**
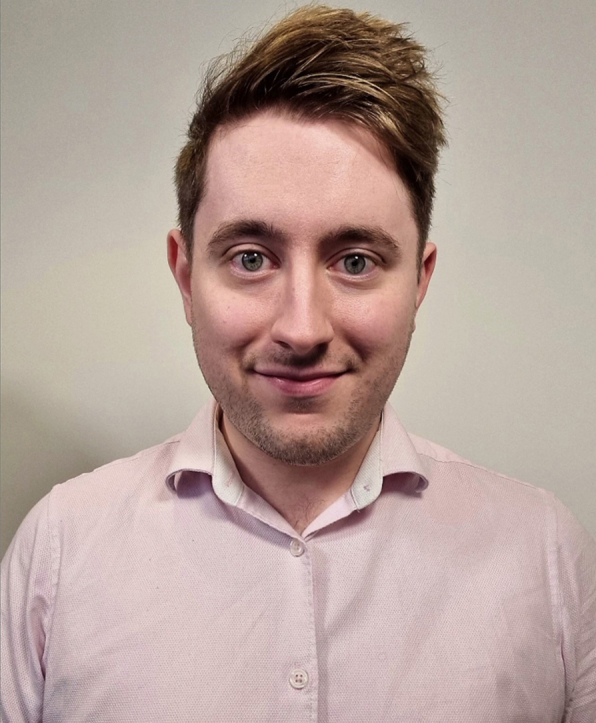

The premise of the accompanying case is deceptively simple: a young woman enters the trauma bay with a penetrating chest injury, and an intracardiac abnormality is identified in a setting where every finding is initially assumed to belong to the emergency. That reflex is useful for resuscitation, but it can mislead interpretation. Bravo et al^1^ show that the more important question is not merely “what is the mass?” but whether it is traumatic, hemodynamically relevant, and likely to benefit from intervention. The mitral valve blood cyst is rare; the lesson is not. Cardiac incidentalomas are increasingly encountered because contemporary acute-care imaging is both highly sensitive and anatomically comprehensive. Their safe management depends on disciplined escalation from detection to localization, tissue characterization, and surveillance.

Blood cysts of the cardiac valves occupy a narrow space between a congenital remnant, a benign tumor mimic, and a valvular red herring. They are most common in fetuses and infants, may regress, and are uncommon but well documented in adults, where mitral involvement predominates.^2^^,^^3^ Although their natural history is usually benign, adult mitral blood cysts should not be dismissed reflexively: reported complications include mitral dysfunction, embolization, and dynamic left ventricular outflow obstruction with syncope.^4^ The diagnostic approach should therefore be probabilistic, anchored in attachment site, morphology, hemodynamic consequence, vascularity, and enhancement.

The authors’ strength lies in correctly sequencing the question. Computed tomography (CT) raised concern for a left ventricular abnormality in a patient whose clinical context made traumatic injury the default assumption. Transthoracic echocardiography narrowed that concern by identifying a hypoechoic mass in the region of the anterior mitral valve leaflet and subvalvular apparatus, while also showing preserved biventricular function and no pericardial effusion. Transesophageal echocardiography then refined anatomical assessment, showing a smooth, thin-walled, centrally echolucent lesion attached to the ventricular aspect of A2/A3, moving with the anterior mitral leaflet and producing no meaningful regurgitation or obstruction. The remit of cardiac magnetic resonance (CMR) was to shift evaluation from anatomical localization toward tissue characterization. Cine steady-state free precession confirmed leaflet attachment, T1 and T2 black-blood imaging suggested proteinaceous or blood-containing cystic content, fat-suppressed imaging showed no signal loss, first-pass perfusion demonstrated avascularity, and phase-sensitive inversion recovery (PSIR) late gadolinium imaging supported the absence of true enhancement. This was an appropriately multimodal, risk de-escalating approach.

A useful refinement is to frame the CMR component explicitly as a dedicated cardiac mass protocol rather than a standard functional study. Such a protocol is tailored to the lesion, but typically includes mass-targeted cine planes, T1-weighted imaging with and without fat suppression or Dixon fat-water separation, T2-weighted/short tau inversion recovery imaging, susceptibility-sensitive T2∗ when blood products or calcification are relevant, first-pass perfusion, early postcontrast imaging with a thrombus-nulling inversion time, and late gadolinium enhancement (LGE) acquired with both magnitude and PSIR reconstructions. Native T1, T2, and, where feasible, postcontrast T1/extracellular volume mapping can add quantitative tissue description, although small mobile valvular lesions require caution because motion and blood-pool partial volume effects may dominate the measurement.^5^, ^6^, ^7^, ^8^ For a mitral leaflet lesion, systolic imaging through the closed valve, thinner slices, and repeat long inversion-time postcontrast imaging after inversion-time optimization can improve diagnostic confidence.

Table 1 provides a useful diagnostic matrix. Its value lies in cross-sequence pattern recognition, because no isolated signal characteristic is specific: T1 hyperintensity may reflect fat proteinaceous material, subacute blood, melanin, or slow flow. T2 hyperintensity may reflect simple fluid, edema, myxoid matrix, or vascularity. The discriminators are convergence and contraindication across sequences. Fat suppression tests lipid content; first-pass perfusion tests vascularity; early postcontrast imaging with thrombus-nulling inversion time helps separate avascular thrombus from enhancing tissue; and LGE, particularly with PSIR, assesses enhancement related to fibrosis, necrosis, or extracellular expansion. In a large CMR cohort, tumors were more likely than thrombi to show T2 hyperintensity, first-pass perfusion, and LGE, whereas malignant tumors more often showed perfusion and LGE.^6^ More recently, a CMR mass score incorporating morphology, infiltration, pericardial effusion, first-pass perfusion, and heterogeneous enhancement provided a structured method for malignancy prediction.^9^ Quantitative first-pass perfusion and parametric mapping may further refine this risk stratification, but should remain subordinate to morphology and clinical context, particularly for small mobile vascular lesions.^10^^,^^11^

**Table 1:** Multiparametric Cardiac Magnetic Resonance Signal Matrix for Common Cardiac Masses

Entity	Morph	T1w	FS	T2/STIR	FPP	EGE	LGE/PSIR
Blood cyst	Valve cyst	↑/↔	0	↑/±	0	0	MAG↑; PSIR 0/↓
Simple cyst	Cystic	↓/↔	0	↑↑	0	0	0
Fatty mass	Fat/capsule	↑↑	↓↓	↑; STIR↓	0	0	0
Acute thrombus	Mural/chamber	↔/↑	0	±/↑	0	Dark	0/rim
Chronic thrombus	Mural	↓/↔	0	↓	0	Dark	0/rim
Myxoma	Atrial stalk	↔/±	0	↑	±	±/↑	↑het
Fibroelastoma	Valve frond	↔	0	↔/↑	0/±	±	±
Fibroma/rhabdomyoma	Intramural	↔/↓	0	↓/↑	0/±	±	±/↑↑
Vascular tumor	Vascular	↔/↑	0	↑↑	↑↑	↑↑	↑↑
Vegetation/abscess	Valve/ring	↔	0	↑	±	Rim	Rim/↑
Malignancy	Invasive	Het	0	↑/het	↑/het	↑	↑het
Caseous MAC/CAT	Annular/calcific	↔/↑	0	↓	0	0	Rim/0

± = variable; ↑ = high/present; ↓ = low/suppressed; ↔ = isointense; 0 = absent; CAT = calcified amorphous tumor; EGE = early gadolinium enhancement; FPP = first-pass perfusion; het = heterogeneous; LGE = late gadolinium enhancement; MAC = mitral annular calcification; MAG = magnitude; Morph = morphology; PSIR = phase-sensitive inversion recovery; rim = peripheral; FS = fat suppression/Dixon; STIR = short tau inversion recovery; T1w = T1-weighted.

Within this matrix, the accompanying lesion sits convincingly in the benign cystic column. It was leaflet-attached, smooth, noninvasive, nonobstructive, nonperfusing, and nonenhancing on PSIR, with no signal loss on fat suppression and no interval change on CT 4 years later. Although histological confirmation is absent, the convergence of transesophageal echocardiography morphology, multiparametric CMR, and longitudinal behavior is clinically persuasive. The authors should be commended for showing how multimodality imaging can prevent a traumatic red herring from becoming an operative diagnosis. CT detected the abnormality, echocardiography established its valvular attachment, and CMR provided the tissue signature that supported conservative management. The broader lesson is that cardiac incidentalomas in acute care should not be dismissed, but neither should they be overattributed to the emergency at hand. Their safe management depends on moving deliverably from detection to anatomic localization, to tissue characterization, and finally to surveillance.

## Funding support and author disclosures

Dr Garg is a clinical advisor for Neosoft, Pie Medical Imaging, and Medis Medical Imaging and consults for Anteris, Abbott, and Edwards Lifesciences. Dr Grafton-Clarke has reported that he has no relationships relevant to the contents of this paper to disclose.
